# Morphea and Extragenital Lichen Sclerosus Et Atrophicus Overlap

**DOI:** 10.7759/cureus.71176

**Published:** 2024-10-09

**Authors:** Ajay Dodeja, Hetal Karani, Sushil Pande

**Affiliations:** 1 Department of Dermatology, Venereology and Leprology, NKP Salve Institute of Medical Sciences and Research Centre and Lata Mangeshkar Hospital, Nagpur, IND

**Keywords:** dermato-pathology, dermoscopy, lichen sclerosus et atrophicus, morphea, overlap, skin biopsy punch

## Abstract

Lichen sclerosus et atrophicus (LSA) is a chronic inflammatory condition that affects mainly the genital regions but can occur extragenitally. Extragenital LSA occurs far less often than the genitally located variety and in most cases has been reported in females. A 36-year-old male engineer presented with chronic itchy lesions on the right forearm for more than a year's duration. On clinical examination, he had hypopigmented atrophic patches with scaling, alopecia, and follicular plugging. Dermoscopy revealed white structureless areas with telangiectasia, comedo-like openings, and peripheral pigment network. Histopathologically, these findings corroborated the presence of hyperkeratosis, epidermal atrophy, thickened collagen, and chronic inflammation. Overlapping LSA with morphea was under consideration since clinical, dermoscopic, and histopathological features were similar between the two conditions. The diagnostic challenge for both disorders resides in their locality in the form of a spectrum of localised sclerosing disorders. This case clearly highlights the importance of combining clinical, dermoscopic, and histopathological assessments for the proper differentiation of LSA from other sclerosing diseases and thus ensuring proper management and care. This is a report on a case that has a unique dermoscopic presentation and may mimic other disorders, necessitating careful diagnostic consideration.

## Introduction

Lichen sclerosus et atrophicus (LSA) is a chronic, autoimmune, inflammatory condition that is identified by hypopigmented and atrophied skin lesions. LSA usually occurs in the genital region, but it may present elsewhere too. Postmenopausal women and premenarchal girls are the main groups afflicted by LSA. It is believed that extragenital LSA is a rare form of the disorder that mostly affects women. Its incidence, which falls between 0.1% and 0.3% [1], is believed to be underestimated [2]. Extragenital LSA affects around 15% of LSA patients [2]. It is estimated that 6% of LSA patients have only extragenital lesions [2]. Morphea, also known as localised scleroderma, is an uncommon inflammatory connective tissue disease that may progress to involve subcutaneous tissues as well. The co-occurrence of LSA and morphea in a patient raises the possibility that these lesions are part of a spectrum that has comparable etiologic events or pathologic processes [3]. The coexistence of LSA and morphea may be explained by oxidative DNA damage and epigenetic processes. By stimulating the type 1 T helper (Th1) immune response and controlling the production of fibrotic tissue, small endogenous noncoding miR-155 contributes significantly to the pathophysiology of LSA [2]. Wolska-Gawron et al. stated that miR-155 plays a part in the regulation of fibrosis in morphea [4].

While it is extensively documented in the literature that LSA and morphea can develop simultaneously, it is uncommon for both conditions to present in the same lesion [5,6].

## Case presentation

**Figure 1:**
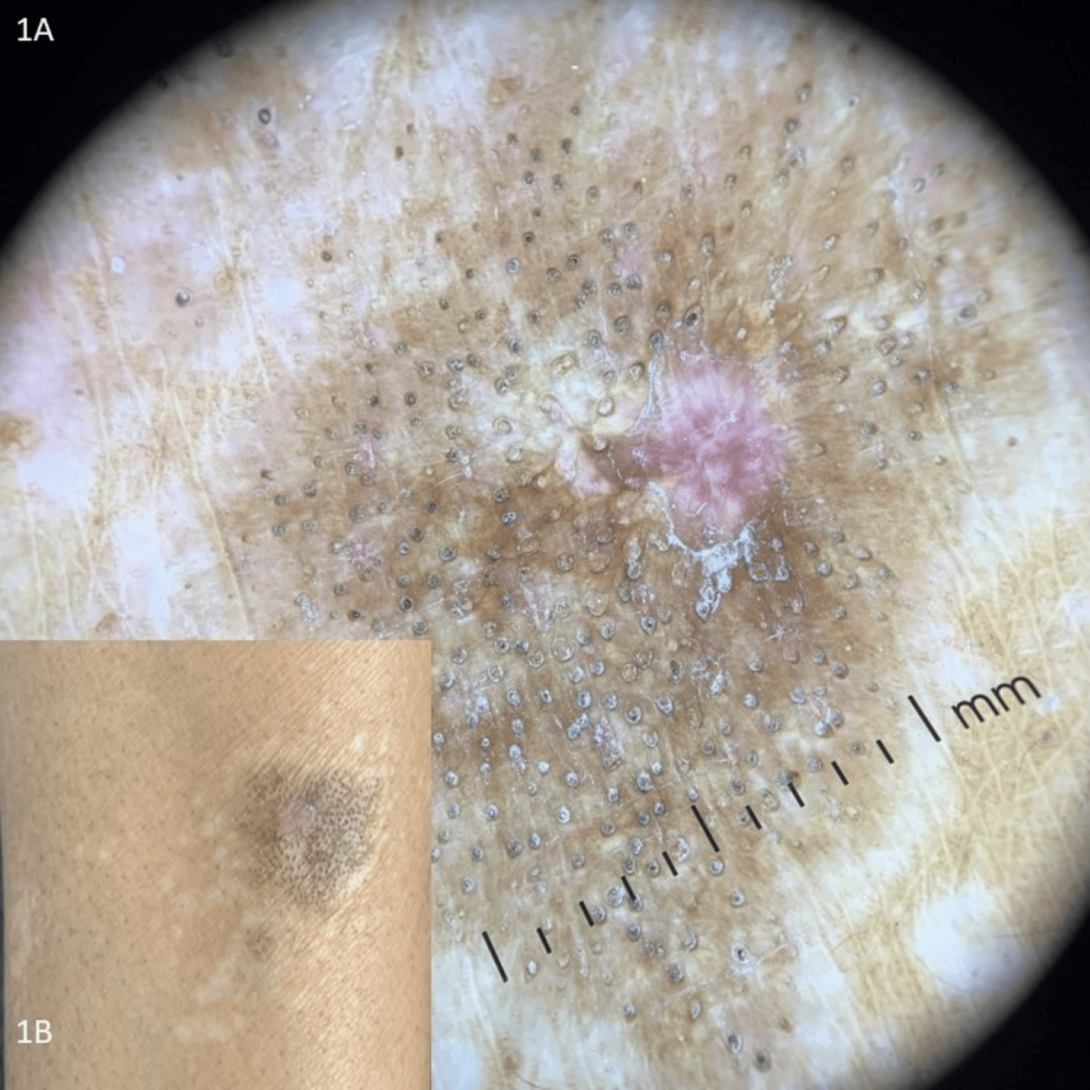
(A) Dermoscopic image showing structureless white areas and comedo-like openings in a peppering pattern (DermLite DL4; 10×). (B) Atrophied hypopigmented patch with scaling and follicular plugging.

**Figure 2:**
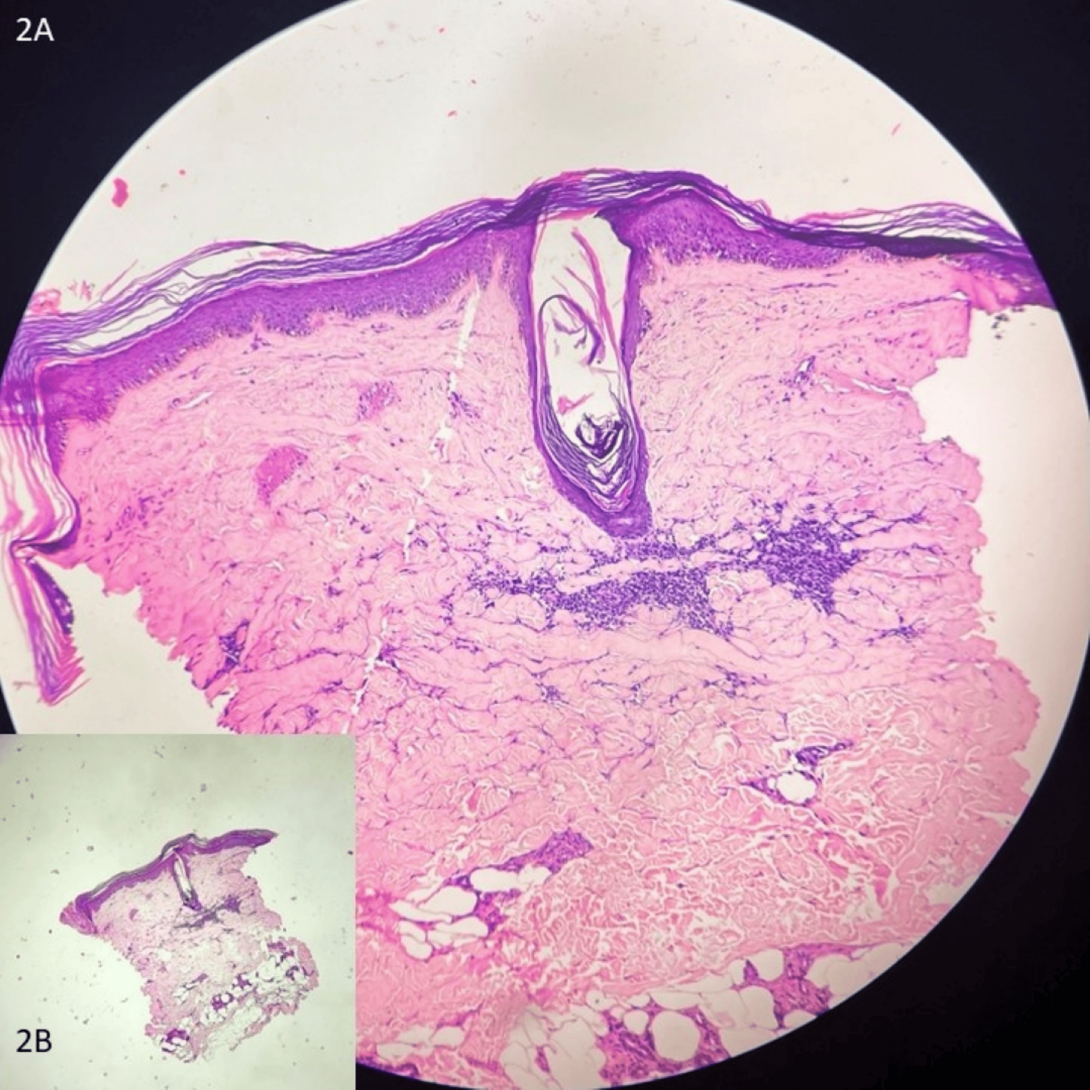
(A) Hyperkeratosis, flattening of rete ridges, focal thinning of the epidermis, and focal degenerative areas in the basal layer. Thickened and widened collagen fibres along with chronic inflammatory infiltrate (H&E; 40×). (B) The squared-off appearance of the biopsy specimen (H&E; 4×). H&E: hematoxylin and eosin

An otherwise healthy 36-year-old male engineer presented with a few itchy lesions over the right forearm persistent for a year. On examination, he had a few well-circumscribed hypopigmented atrophied patches associated with mild scaling, alopecia, and follicular plugging (Figure 1B). Dermoscopy showed white structureless areas intercepted with telangiectasia, milky-red areas, comedo-like openings in a peppering pattern, and a peripheral pigment network (Figure 1A). Histopathological examination revealed hyperkeratosis, flattening of rete ridges, focal thinning of the epidermis, and focal degenerative areas in the basal layer. Thickened and widened collagen fibres along with chronic inflammatory infiltrate were seen at places in the lower dermis (Figure 2A).

Dermoscopic features like white structureless areas, comedo-like openings in a peppering pattern that histopathologically correlate to atrophy in the epidermis and upper dermis, and follicular plugging, respectively, along with degenerative areas of the basal layer were suggestive of LSA, whereas thickened and widened collagen fibres along with chronic inflammatory infiltrate seen at places extending up to the lower dermis and the squared-off appearance of the tissue (Figure 2B) were suggestive of morphea histopathologically, thereby confirming the diagnosis of morphea and LSA overlap.

The patient was prescribed tacrolimus 0.1% ointment to be applied locally at bedtime, along with emollients to be applied twice daily. The lesions have been showing gradual improvement and are being monitored monthly, while the complete resolution of the lesions is awaited.

## Discussion

The most prevalent extragenital locations for LSA are the trunk and extremities. LSA often manifests as white atrophic plaques and papules, which are most frequently observed on the vulvar and perianal areas but can occur elsewhere too [7].

Increased collagen and thicker skin with induration in the lesion are characteristics of morphea. While LSA and morphea are identified as distinct clinical entities, they might also be viewed as disorders at different extremes of the localised sclerosing disorder spectrum. The histopathological features of LSA include homogenised papillary dermis, follicular plugging, hyperkeratosis, epidermal atrophy, and basal cell degeneration. Conversely, morphea is distinguished by perivascular lymphocytes and plasma cells, squared-off biopsies, inflated collagen bundles, confined adnexa, and a heavily sclerotic dermis [6,8]. An overlap between morphea and LSA, resembling lupus vulgaris, has been reported by Prasanna et al. [9] and Kakizaki et al. [8], with dermoscopic findings showing follicular plugging and yellowish-brown crusts, underscoring the challenges in differential diagnosis.

Immunohistochemical similarities between LSA and morphea have been demonstrated in a case study, highlighting the complexity of these conditions [8]. Immunohistochemical staining for periostin, matrix metalloproteinase-7 (MMP-7), and MMP-28, known to facilitate fibrosis in various organs including the skin, was utilized in a case of LSA accompanied by morphea, providing insights into potential mechanisms underlying the fibrotic processes in these disorders [8].

The importance of recognizing the varied dermoscopic features of LSA, which can mimic other dermatological conditions, is emphasized [10]. Accurate diagnosis and differentiation are crucial due to the risks of atrophy, scar formation, and malignant changes associated with LSA [11]. Furthermore, the overlap between LSA and other conditions like morphea underscores the necessity of a comprehensive approach involving clinical, dermoscopic, and histopathological assessments for precise diagnosis and management [8].

## Conclusions

Dermoscopic features like white structureless areas, comedo-like openings in a peppering pattern that histopathologically correlate to atrophy in the epidermis and upper dermis, and follicular plugging, respectively, along with degenerative areas of the basal layer were suggestive of LSA, whereas thickened and widened collagen fibres along with chronic inflammatory infiltrate seen at places extending up to the lower dermis and the squared-off appearance of the tissue were suggestive of morphea histopathologically.

We report a case that was diagnosed as LSA dermoscopically and turned out to be morphea LSA overlap on clinico-dermoscopic-histopathological correlation. Our experience demonstrates that, by integrating clinical findings, dermoscopy, and histopathology, dermatologists can effectively navigate the complexities of LSA and its potential overlaps with other sclerosing disorders, ensuring appropriate management and care for affected individuals.
